# Transesophageal echocardiography-guided anesthetic management of a patient with dilated cardiomyopathy, severe heart failure, and septic shock: a case report

**DOI:** 10.3389/fmed.2026.1853263

**Published:** 2026-06-29

**Authors:** Lixin Chen, Jianbin Li, Mintai Gao, Danjun Zhu, Ruiyu Li, Ganghua Yang

**Affiliations:** 1 Xiaolan Clinical Institute of Shantou University Medical College, Zhongshan, China; 2 Department of Anesthesiology, Xiaolan People's Hospital of Zhongshan (The Fifth People's Hospital of Zhongshan), Zhongshan, China

**Keywords:** anesthetic management, dilated cardiomyopathy, heart failure, perioperative care, septic shock, transesophageal echocardiography

## Abstract

Perioperative management of severe dilated cardiomyopathy (DCM) with markedly reduced left ventricular ejection fraction (LVEF) is particularly complex, especially when complicated by septic shock, as standard resuscitative measures may trigger acute cardiac decompensation. This report describes a 41-year-old male with undiagnosed severe DCM (LVEF 16% by transthoracic echocardiography) and left ventricular thrombus, who presented with Aeromonas hydrophila-induced necrotizing fasciitis that rapidly progressed to septic shock and multiple organ dysfunction. The severity of septic manifestations obscured the underlying occult cardiomyopathy, underscoring the critical role of cardiac biomarkers and echocardiography in facilitating early diagnosis. Intraoperative transesophageal echocardiography (TEE) proved pivotal for the real-time, physiology-guided titration of a multi-agent pharmacological regimen (norepinephrine, dobutamine, esmolol) and for guiding positional adjustments, thereby balancing the conflicting hemodynamic demands of septic vasoplegia and end-stage heart failure during emergent limb-saving surgery.

## Introduction

Dilated cardiomyopathy (DCM) is a primary myocardial disorder characterized by biventricular or left ventricular dilation and systolic dysfunction, often progressing to severe heart failure (HF) (1). Managing DCM patients with profoundly reduced left ventricular ejection fraction (LVEF) is challenging due to the fragile balance between preload dependency and volume overload risk, compounded by a high propensity for life-threatening tachyarrhythmias (2). Concomitant septic shock dramatically compounds the clinical complexity: septic vasoplegia, myocardial depression, and altered vascular permeability impose severe hemodynamic burdens, with conventional resuscitation potentially inducing acute decompensation of the underlying cardiomyopathy (3). Infection source control often requires urgent surgery, but anesthesia for non-cardiac surgery in this cohort carries extremely high perioperative mortality driven by anesthetic-induced myocardial depression, perioperative fluid shifts, and surgical stress on a sepsis-compromised failing heart (4). Standard hemodynamic monitoring fails to strike a balance between maintaining adequate perfusion and protecting ventricular function, creating a need for precision-guided anesthetic management. To address this unmet need, we propose a novel TEE-guided stratification strategy centered on LVEF—the core indicator of cardiac reserve—for DCM patients complicated by septic shock (Table 1).

**Table 1:** LVEF-stratified TEE-guided hemodynamic management for high-risk perioperative patients.

LVEF stratification	TEE monitoring focus	Core hemodynamic strategy	MCS indication
>35% (mild)	Volume status, IVC collapsibility	Moderate goal-directed fluid resuscitation, low-dose vasopressors	Persistent shock despite optimal therapy
20%−35% (moderate)	Global function, CO/SV, preload	Judicious fluid resuscitation, positional preload reduction, vasopressor + inotrope	Refractory hypotension/HF decompensation
< 20% (end-stage)	Global hypokinesis, thrombus stability, biventricular function	Strict volume restriction, positional preload reduction, low-dose vasopressors	Severe cardiogenic shock (lactate >4 mmol/L)

MCS, mechanical circulatory support; IVC, inferior vena cava; CO, cardiac output; SV, stroke volume.

This report details the perioperative anesthetic management of a 41-year-old male with previously undiagnosed severe DCM (LVEF 16%; classified as end-stage) and left ventricular thrombus, who presented with Aeromonas hydrophila necrotizing fasciitis progressing to septic shock (5). This case supports the proposed stratified strategy.

## Case presentation

### Patient information

A 41-year-old married male from Wuzhou, Guangxi, was admitted on March 5, 2026, with 1 day of right lower limb redness, swelling, and exudation. The onset was insidious with progressive symptoms; he had no pain, dizziness, fever, or other complaints. Past medical history was unremarkable (no coronary heart disease, hypertension, diabetes, surgery, or allergy history); he had no smoking/alcohol consumption and no toxin/epidemic exposure. Family history was non-contributory. He was provisionally diagnosed with infectious fasciitis. Baseline patient characteristics are summarized in Table 2.

**Table 2:** Baseline patient characteristics.

Characteristic	Value
Age (years)	41
Sex	Male
Body mass index (kg/m^2^)	23.4
Past medical history	None (no coronary artery disease, hypertension, diabetes, prior surgery, or allergies)
Smoking/Alcohol	None
Presenting symptom	Right lower limb redness, swelling, exudation for 1 day
Admission diagnosis	Necrotizing fasciitis (right lower limb)
Pathogen	Aeromonas hydrophila
Admission vital signs	BP 82/46 mmHg (MAP 58 mmHg), HR 150 bpm (atrial flutter), RR 28/min, temperature 38.9 °C, SpO_2_ 92% (FiO_2_ 0.5)
Laboratory findings	Pro-BNP 12,140.5 pg/mL, high-sensitivity troponin T elevated, CRP 214.0 mg/L, lactate 4.6 mmol/L
Transthoracic echocardiography (admission)	LVEF 16%, four-chamber enlargement, left ventricular apical thrombus (29 × 25 mm)
Intraoperative TEE (initial)	LVEF 20%, thrombus stable
Other findings	Right great saphenous vein thrombosis, acute kidney injury, hypoalbuminemia

### Clinical findings

On admission, the patient was intubated, sedated and mechanically ventilated. Vital signs were as follows: blood pressure 82/46 mmHg [mean arterial pressure (MAP) 58 mmHg], heart rate 150 beats/min (irregular, atrial flutter), respiratory rate 28 breaths/min, tympanic temperature 38.9 °C, and peripheral oxygen saturation (SpO_2_) 92% on mechanical ventilation (FiO_2_ 0.5). He was drowsy with a Richmond Agitation-Sedation Scale (RASS) score of −3 (moderate sedation). No jaundice, pallor, or cyanosis was observed. Cardiovascular examination revealed tachycardia with an irregular rhythm; no obvious valvular murmurs, pericardial friction rub, or gallop rhythm were auscultated. Respiratory examination showed bilateral coarse crackles at the lung bases, consistent with mild pulmonary congestion. Abdominal examination was soft, non-tender, with no hepatosplenomegaly. Lower extremity examination demonstrated extensive erythema, blistering, severe swelling, and copious serosanguineous wound exudate from the right ankle to the inguinal region/groin, with minimal bleeding. Distal pulses were weakly palpable. Skin necrosis, subcutaneous crepitus, and right great saphenous vein thrombosis were noted upon surgical debridement. No pitting edema was observed in the left lower extremity. Initial laboratory tests showed Pro-BNP 12,140.5 pg/mL; transthoracic echocardiography revealed four-chamber enlargement, LVEF 16%, and a left ventricular apical thrombus (29 × 25 mm). Chest radiography demonstrated increased lung markings and cardiomegaly. Postoperatively, the patient was transferred to the ICU with persistent tachycardia, requiring vasopressor and antiarrhythmic support. Wound culture confirmed Aeromonas hydrophila as the pathogen.

### Diagnostic assessment

**Figure 1:**
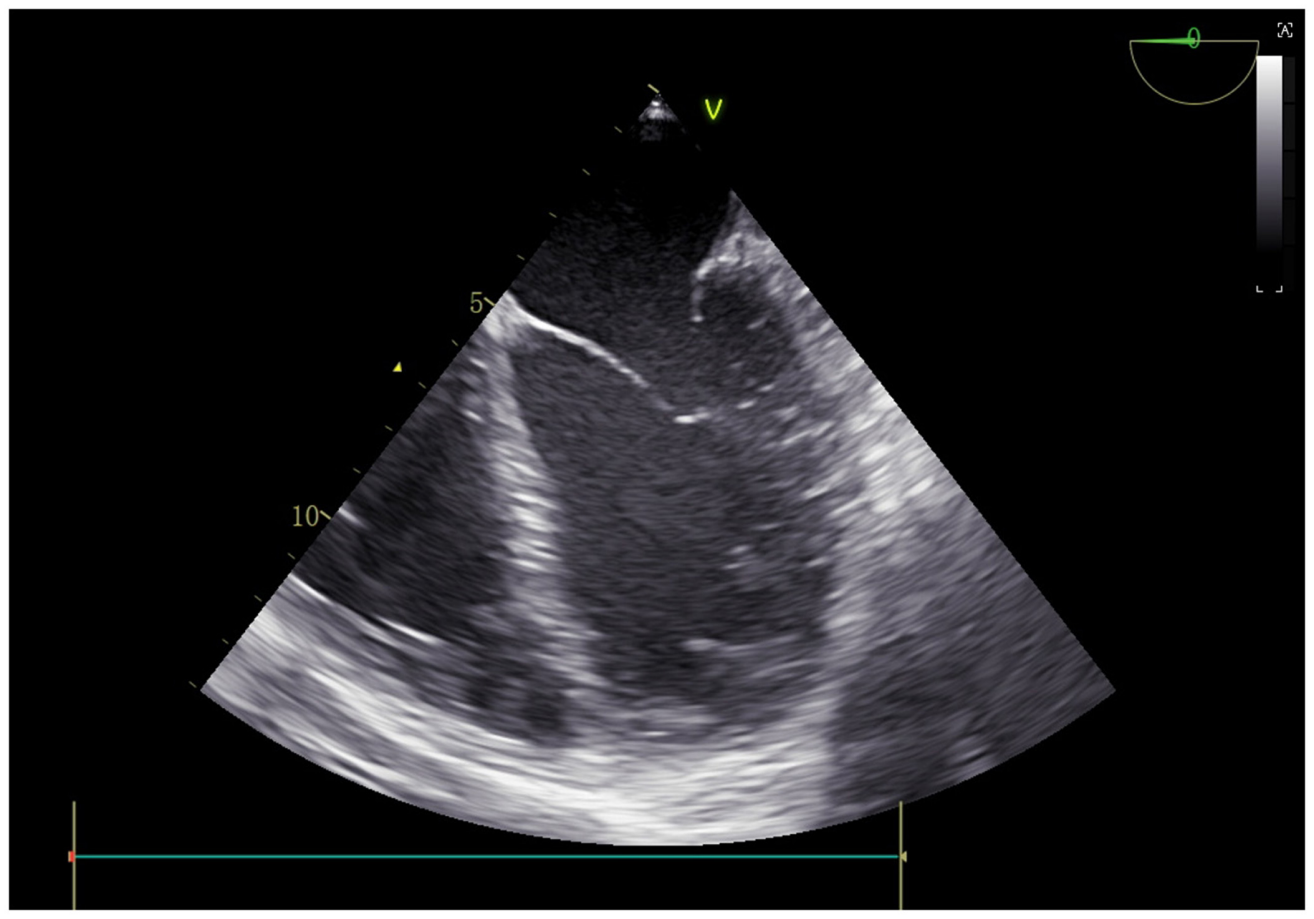
Mid-esophageal 4-chamber view.

**Figure 2:**
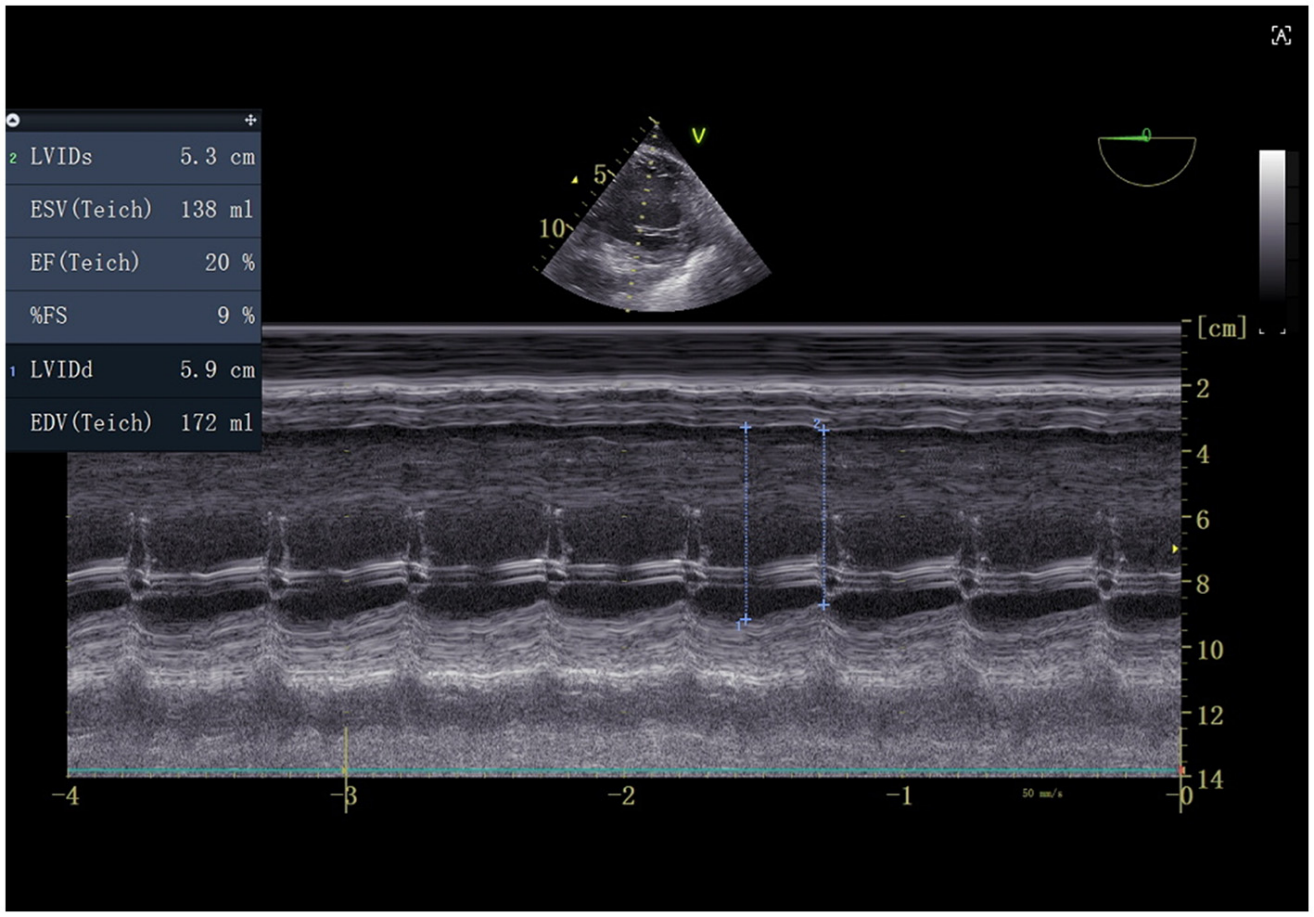
Mid-esophageal short-axis M-mode view.

The patient was diagnosed with necrotizing fasciitis of the right lower limb induced by Aeromonas hydrophila, septic shock, and multiple organ dysfunction syndrome (MODS) involving the circulatory, renal, coagulation, and cardiac systems. Evaluation of cardiac function revealed severe DCM with HF [cardiomegaly, global left ventricular systolic dysfunction (characterized by an LVEF of 16% by transthoracic echocardiography and 20% by intraoperative TEE), representative intraoperative TEE images are shown in Figures 1 and 2 left ventricular apical thrombus], accompanied by right great saphenous vein thrombosis, hypoalbuminemia, and acute kidney injury. Serial laboratory tests confirmed persistent infection (leukocytosis, elevated CRP and procalcitonin) and myocardial injury (elevated high-sensitivity troponin T).

### Therapeutic intervention

Initial management focused on urgent surgical debridement and intraoperative broad-spectrum antibiotics (piperacillin-tazobactam + clindamycin), later escalated to imipenem-cilastatin + levofloxacin. For septic shock and severe HF, the patient received continuous norepinephrine (0.3 μg/kg/min initially), dobutamine, and amiodarone + esmolol for atrial flutter. Anticoagulation was initiated for left ventricular thrombus, and continuous renal replacement therapy (CRRT) was used for acute kidney injury.

Serial lactate measurements were obtained as part of the hemodynamic management strategy. On admission, the arterial lactate level was 4.6 mmol/L. After initial resuscitation in the ICU, the lactate decreased to 3.1 mmol/L on the morning of surgery (just before anesthetic induction).

On March 8, the patient was transferred to the operating room for further debridement. Before anesthetic induction, his vital signs were: blood pressure 96/52 mmHg (MAP ~67 mmHg), heart rate 148 bpm (atrial flutter), respiratory rate 24 breaths/min (ventilator-controlled), SpO_2_ 96% on FiO_2_ 0.4, and RASS −2. Guided by the end-stage LVEF stratification (Table 2), perioperative monitoring focused on global hypokinesis, thrombus stability, and biventricular function via TEE. TEE probe specifications are provided in Table 3. The standardized TEE imaging planes used are summarized in Table 4.

**Table 3:** TEE probe specifications and technical parameters.

Parameter	Specification
Manufacturer	GE Healthcare
Model	6VT-D TEE probe (compatible with Vivid E95 or E90 ultrasound system)
Frequency range	3–8 MHz (broadband, fully sampled matrix array)
Imaging modes	2D, M-mode, color doppler, pulse-wave doppler, continuous-wave doppler, 3D/4D (Live 3D TEE)
Thermal index (TI)	< 1.0 (during intraoperative use)
Mechanical index (MI)	0.7–1.0 (adjusted per clinical need)
Probe dimensions	Tip dimensions: 15.2 mm × 10.5 mm × 8.2 mm; shaft length: 110 cm; bending section: two-way (anterior/posterior) 120°, lateral 90°
Sterilization method	High-level disinfection with peracetic acid (e.g., Cidex OPA)

**Table 4:** Intraoperative TEE imaging planes.

Imaging plane	Probe position	Multiplane angle	Primary clinical target
Transgastric short-axis (mid-papillary)	Transgastric (40–45 cm from incisors), anteflexed	0°	Real-time LVEF, regional wall motion, cavity dimensions
Mid-esophageal four-chamber	Mid-esophagus (30–35 cm)	0°–20°	LV and RV size/function, thrombus stability (apical), biventricular interaction
Mid-esophageal two-chamber	Mid-esophagus	90°	Orthogonal LVEF assessment, anterior/inferior wall motion
Mid-esophageal long-axis	Mid-esophagus	120°–140°	LVOT patency, aortic valve morphology, thrombus extension
Modified mid-esophageal bicaval (IVC view)	Mid-esophagus, rightward rotation	90°–110°	Volume status (IVC collapsibility; < 15% indicated adequate preload)

MAP, mean arterial pressure; HR, heart rate; NE, norepinephrine; DB, dobutamine; ES, esmolol; AM, amiodarone; TEE, transesophageal echocardiography; ICU, intensive care unit; LVEF, left ventricular ejection fraction; LV thrombus, left ventricular thrombus; CRRT, continuous renal replacement therapy.

Additional monitoring included radial artery invasive blood pressure and CVP. Arterial blood gas, lactate, and electrolytes were monitored every 30 min, with targets of lactate < 2.0 mmol/L, K+ 3.5–4.5 mmol/L, and CVP 10–15 cmH_2_O.

Anesthetic induction was performed using a cardiac-sparing regimen consisting of sufentanil 10 μg, etomidate 12 mg, and cisatracurium 10 mg, followed by uneventful video laryngoscopy intubation. Intraoperative hemodynamic optimization included strict volume restriction (200 mL lactated Ringer's solution) and 20° head-up tilt (reducing CVP from 25 to 18 mmHg). Multi-target pharmacotherapy was titrated as follows: dobutamine 2.0–5.0 μg/kg/min (q5min adjustments), esmolol 50–200 μg/kg/min (maintaining ventricular rate 80–100 bpm), amiodarone 0.5–1.0 mg/min, and norepinephrine (titrated for MAP 65–80 mmHg). The 1.5-h procedure resulted in 50 mL of blood loss and 50 mL of urine output, with hemodynamics remaining stable throughout. At the end of surgery, the arterial lactate was 1.7 mmol/L, meeting the target of < 2.0 mmol/L. The full serial perioperative hemodynamic indicators from admission to ICU day 5 are summarized in Table 5.

**Table 5:** Perioperative clinical timeline from admission to ICU Day 5.

Time point	MAP (mmHg).	HR (bpm)	Vasoactive drugs (μg/kg/min)	Cumulative fluid balance (mL)	Lactate (mmol/L)	Key TEE findings
Admission (Day 1)	55–62	145–155 (atrial flutter)	NE 0.3	+800 (initial resuscitation)	4.6	TTE: LVEF 16%, LV thrombus 29 × 25 mm
Preoperative morning (Day2, before induction)	60–66	135–145	NE 0.2, DB 2.0	+1,200	3.1	–
Intraoperative core period (0.5 h)	65–80	80–100	NE 0.2–0.3, DB 2.0–5.0, ES 50–200, AM 0.5–1.0 mg/min	+200 (total intraop fluid)	1.7	TEE: LVEF 20%, thrombus stable
End of surgery	70–80	85–95	NE 0.2, DB 3.0	+200	1.7	TEE: LVEF 20%, thrombus stable
ICU Day 1 (postop Day 0–1)	65–75	90–100	NE 0.2, DB 3.0	CRRT-guided negative balance	1.6	–
ICU Days 2–5	70–80	85–95	NE weaned to 0.05–0.1, DB 2.0–3.0	Progressive negative balance	1.2–1.5	Repeat TTE at Day 5: LVEF 22%, thrombus stable

Postoperatively, TEE confirmed LVEF 20% with no thrombus displacement; the patient was transferred to the ICU intubated, with continued norepinephrine 0.2 μg/kg/min + dobutamine 3.0 μg/kg/min, and ventilator settings of volume-controlled ventilation (6 mL/kg tidal volume, 5 cmH_2_O PEEP, 40% FiO_2_).

### Follow-up and outcomes

The timeline of key clinical events and interventions is presented in Table 6. ICU monitoring showed improved local infection (reduced wound exudate, CRP 214.0 → 82.5 mg/L), but persistent atrial flutter required cardiology consultation. Hemodynamics stabilized with reduced vasopressor requirements; CRRT-guided negative fluid balance relieved lower limb edema. The patient was weaned to high-flow oxygen therapy with SpO_2_ 98%−100%. He was discharged from the ICU on postoperative day 10 and from the hospital on day 21 without major adverse cardiac events.

**Table 6:** Timeline of key clinical events, interventions and outcomes.

Time point	Key clinical events and interventions
Day 1 (Admission)	Right lower limb necrotizing fasciitis; rapidly progressed to septic shock; intubation and mechanical ventilation; initial resuscitation; TTE showed severe dilated cardiomyopathy (LVEF 16%) and left ventricular apical thrombus; elevated Pro-BNP, troponin, lactate, CRP and procalcitonin; broad-spectrum antibiotics initiated; CRRT for acute kidney injury
Day 2 (Preoperative)	Hemodynamic stabilization with norepinephrine, dobutamine, amiodarone and esmolol; lactate decreased to 3.1 mmol/L; ongoing infection control and anticoagulation
Day 3 (Intraoperative)	Emergency debridement under TEE-guided anesthesia; cardiac-sparing induction; strict fluid restriction; 20° head-up tilt; TEE monitoring of biventricular function, thrombus stability and volume status; targeted vasoactive drug titration; lactate normalized to 1.7 mmol/L; hemodynamically stable
Postoperative Days 1–5	ICU admission; continued inotropic and vasopressor support; CRRT-guided negative fluid balance; gradual weaning of vasoactive agents; improved wound healing; TTE on Day 5 revealed LVEF 22% with stable thrombus
Hospital day 21	Patient discharged home in stable condition without major adverse cardiac events

All views were obtained with care to avoid excessive probe manipulation given the presence of left ventricular thrombus.

### Long-term follow-up

The patient was followed for 1 month after discharge. He remained alive at the last follow-up (May 2026). Serial transthoracic echocardiography (TTE) was performed at 1 month. 1 month post-discharge: LVEF improved to 28% (from 16% on admission). The left ventricular apical thrombus (29 × 25 mm on initial imaging) had decreased in size to 18 × 15 mm with no evidence of embolization. Anticoagulation with warfarin (target INR 2.0–3.0) was continued.

Guideline-directed medical therapy (GDMT) for heart failure with reduced ejection fraction was initiated during the index hospitalization and optimized at the cardiology outpatient clinic. The patient was prescribed bisoprolol (target dose 10 mg daily, achieved 7.5 mg), sacubitril/valsartan (97/103 mg twice daily), spironolactone (25 mg daily), and empagliflozin (10 mg daily). Cardiology consultation confirmed that the patient had been referred to a tertiary heart failure center for further evaluation for advanced therapies.

### Patient perspective

After the patient regained full consciousness, the clinical team discussed the perioperative events and long-term treatment goals with him and his wife. The patient expressed that his primary goal was to “return to work and support his family” (he was the sole income earner).

He understood the high risk of recurrent heart failure decompensation and sudden cardiac death and agreed to adhere to lifelong medication and regular follow-up. His wife stated that “we trust the doctors' decisions” and wanted all feasible lifesaving measures to be taken during the acute phase. These goals were documented in the medical record and guided the escalation of heart failure therapy.

## Discussion

Anesthetic management of DCM patients undergoing non-cardiac surgery is inherently high-risk, especially with septic shock. The coexistence of undiagnosed severe systolic dysfunction (LVEF 16%−20%) and fulminant Aeromonas hydrophila necrotizing fasciitis is extremely rare (6). Sepsis-induced cardiomyopathy (SICM) is reversible (7), but this case involved pre-existing chronic DCM, creating a “double-hit” pathophysiology where intrinsic pump failure and septic vasoplegia synergistically threatened circulatory collapse (8). This distinction is critical: SICM management focuses on supportive care, while chronic severe DCM requires minimizing myocardial depression, avoiding volume overload, and balancing perfusion and myocardial oxygen consumption.

In this case, the markedly elevated high-sensitivity troponin T and Pro-BNP raised the differential diagnosis among three entities: (i) pre-existing chronic DCM, (ii) septic cardiomyopathy (SICM), and (iii) supply-demand mismatch-induced myocardial ischemia. Transthoracic and intraoperative transesophageal echocardiography (TEE) played a decisive role in the differentiation. The presence of four-chamber enlargement, a severely reduced LVEF (16%−20%), and a left ventricular apical thrombus on initial imaging were inconsistent with pure SICM, which typically shows reversible regional or global dysfunction without pre-existing structural abnormalities. Supply-demand mismatch due to tachycardia and hypotension could contribute to troponin release, but the chronic biventricular dilation and the absence of regional wall motion abnormalities (e.g., no segmental dyskinesis or thinning) argued against acute coronary syndrome. TEE further excluded apical ballooning characteristic of stress cardiomyopathy (Takotsubo), which usually spares the base and presents with hyperdynamic basal segments–a pattern not observed in this patient. Thus, the combination of structural echocardiographic features and biomarker elevation confirmed the diagnosis of acute-on-chronic heart failure precipitated by septic shock, rather than primary ischemic or stress-induced injury.

Our proposed TEE-guided stratification strategy addresses this conflict by individualizing management based on LVEF. For end-stage DCM, TEE monitoring prioritizes global hypokinesis, thrombus stability, and biventricular function, with core strategies of strict volume restriction and positional preload reduction—exactly the approach that enabled successful hemodynamic control. This framework resolves the therapeutic paradox of septic shock and end-stage HF (9), as demonstrated by precise titration of dobutamine and esmolol. Notably, the strategy's emphasis on “continuous TEE monitoring + restrictive volume management” aligns with the “Chinese Experts' Clinical Management Consensus on the Peri-Anesthesia Period of Non-Cardiac Surgery in Patients with Heart Disease (2020)” which recommends goal-directed hemodynamic management for high-risk cardiac patients (10). Left ventricular thrombus added complexity, requiring a balance between anticoagulation and bleeding risk (11). We combined preoperative anticoagulation with intra-operative measures (avoiding excessive heparin, maintaining stable hemodynamics, and ensuring careful hemostasis) to mitigate risk (12). Aeromonas hydrophila is a virulent pathogen, underscoring the need for anesthesiologists to screen for occult cardiac disease in septic shock patients with such pathogens.

The combination of amiodarone and esmolol in this patient warrants specific justification. The patient presented with persistent atrial flutter at a ventricular rate of approximately 150 bpm, which contributed to reduced diastolic filling time, increased myocardial oxygen consumption, and compromised coronary perfusion—all particularly deleterious in end-stage DCM. Esmolol, an ultra-short-acting β1-selective blocker, was chosen for rapid rate control (target 80–100 bpm) with a short context-sensitive half-life (approximately 9 min), allowing minute-to-minute titration guided by TEE and invasive hemodynamics.

The rationale for esmolol use in septic shock is supported by the landmark open-label randomized phase 2 trial by Morelli et al. (13), which demonstrated that titrated esmolol (to maintain heart rate 80–94 bpm) in patients with severe septic shock (heart rate ≥95 bpm, requiring high-dose norepinephrine) was associated with significant reductions in heart rate, norepinephrine requirements, and fluid balance, together with an improvement in stroke volume index and left ventricular stroke work index. Importantly, these benefits were achieved without an increase in adverse events, and the esmolol group showed a lower 28-day mortality (49.4 %) compared with the control group (80.5 %) (13). However, that trial excluded patients with baseline LVEF below 25 %, and none of the enrolled patients had pre-existing chronic dilated cardiomyopathy. Therefore, the extrapolation of these findings to a patient with end-stage DCM (LVEF 16 %) remains speculative, and our use of esmolol was consequently guided by continuous TEE monitoring of ventricular function.

Esmolol carries a dose-dependent negative inotropic effect. To mitigate the risk of worsening cardiac output, we simultaneously infused dobutamine (2–5 μg/kg/min), providing positive inotropy to offset esmolol-induced depression. This “inotrope-β-blocker” balance was monitored in real time by TEE-derived LVEF and stroke volume. Amiodarone was added not primarily for acute rate control (which was achieved with esmolol) but for its class III antiarrhythmic properties to maintain sinus rhythm and prevent relapse of atrial flutter after rate control. In addition, amiodarone has a favorable hemodynamic profile with minimal negative inotropy when administered intravenously at the dose used (0.5–1.0 mg/min), making it safer than other antiarrhythmics (e.g., flecainide or propafenone) in severe systolic dysfunction. The combined regimen, although carrying a theoretical risk of additive negative inotropy, was successfully implemented under continuous TEE guidance, and no hemodynamic deterioration was observed. We emphasize that this strategy should be reserved for patients with refractory rapid atrial arrhythmias where heart rate control outweighs the potential risks, and it requires advanced monitoring and expertise.

This case highlights key teaching points: severe septic manifestations can mask asymptomatic cardiomyopathy, and elevated Pro-BNP is a critical red flag (14), (15). Anesthesiologists must perform proactive cardiac assessments (biomarkers + echocardiography) for septic shock patients with unexplained hemodynamic instability (16).

Pathophysiologically, the DCM heart (flat Frank-Starling curve) is preload-insensitive and inotropy-dependent (17), while septic shock induces vasoplegia, inflammatory myocardial depression, and tachycardia (18). Our TEE-guided management employed a cardiac-sparing induction regimen (etomidate) and multi-target pharmacotherapy: norepinephrine for coronary perfusion, dobutamine for inotropy, and esmolol for rate control (19).

In conclusion, this case supports the feasibility of the proposed TEE-guided LVEF stratification strategy for DCM complicated by septic shock. Core strategies include pre-anesthetic identification of occult cardiac dysfunction, cardiac-sparing induction, real-time TEE-guided interventions tailored to stratification, and multi-modal pharmacotherapy. The findings of this case advocate for integrating this approach into clinical practice for high-risk patients, in line with guidelines (20).

This report has several limitations. As a single-center, single-case report, the generalizability of the proposed stratified strategy is limited, and multi-center validation is needed. Anesthetic management relied on clinical experience rather than standardized TEE-guided drug titration, and the dose of dobutamine was limited due to the presence of left ventricular thrombus. Cerebral oxygen saturation monitoring was not performed. Successful management depended on advanced TEE expertise and multidisciplinary collaboration. In addition, three specific limitations warrant emphasis. First, the evidence supporting the use of esmolol in septic shock has been derived exclusively from patients without pre-existing severe cardiomyopathy; extrapolating this evidence to a patient with an LVEF of only 16% carries a significant evidence gap. Second, pulmonary artery catheter data were not obtained. Consequently, the hemodynamic targets based on central venous pressure (CVP 10–15 cmH_2_O) may not accurately reflect true cardiac filling pressures in the presence of right-ventricular-to-left-ventricular interaction. Third, although the proposed LVEF-stratified TEE management framework (Table 3) is clinically logical, it does not specify the intraoperative intervals for repeated TEE examinations–an essential operational detail for bedside implementation. Future iterations of the protocol should define clear timing for TEE reassessment (e.g., after induction, with significant hemodynamic changes, after positional adjustments, every 30 min, and at the end of surgery). Further multi-center studies are required to establish the broader efficacy of this stratified strategy.

## Data Availability

The original contributions presented in the study are included in the article/supplementary material, further inquiries can be directed to the corresponding author.
